# Metaverse Wearables for Immersive Digital Healthcare: A Review

**DOI:** 10.1002/advs.202303234

**Published:** 2023-09-22

**Authors:** Kisoo Kim, Hyosill Yang, Jihun Lee, Won Gu Lee

**Affiliations:** ^1^ Intelligent Optical Module Research Center Korea Photonics Technology Institute (KOPTI) Gwangju 61007 Republic of Korea; ^2^ Department of Nursing College of Nursing Science Kyung Hee University Seoul 02447 Republic of Korea; ^3^ Department of Mechanical Engineering College of Engineering Kyung Hee University Yongin 17104 Republic of Korea

**Keywords:** digital healthcare, extended reality, medical twin, metaverse wearables, post‐pandemic

## Abstract

The recent exponential growth of metaverse technology has been instrumental in reshaping a myriad of sectors, not least digital healthcare. This comprehensive review critically examines the landscape and future applications of metaverse wearables toward immersive digital healthcare. The key technologies and advancements that have spearheaded the metamorphosis of metaverse wearables are categorized, encapsulating all‐encompassed extended reality, such as virtual reality, augmented reality, mixed reality, and other haptic feedback systems. Moreover, the fundamentals of their deployment in assistive healthcare (especially for rehabilitation), medical and nursing education, and remote patient management and treatment are investigated. The potential benefits of integrating metaverse wearables into healthcare paradigms are multifold, encompassing improved patient prognosis, enhanced accessibility to high‐quality care, and high standards of practitioner instruction. Nevertheless, these technologies are not without their inherent challenges and untapped opportunities, which span privacy protection, data safeguarding, and innovation in artificial intelligence. In summary, future research trajectories and potential advancements to circumvent these hurdles are also discussed, further augmenting the incorporation of metaverse wearables within healthcare infrastructures in the post‐pandemic era.

## Graphical abstract

This review critically examines the landscape and future applications of metaverse wearables toward immersive digital healthcare. Metaverse wearables, especially coupled with XR technology, will contribute to realizing a paradigm shift in everyday life by being applied to various healthcare fields, such as personalized occupational, educational, and home healthcare applications in the post‐pandemic era.

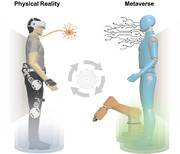

## 1 Introduction

The advent of the metaverse has heralded a new era of digital experiences, blurring the lines between the physical and virtual worlds.^[^ ^1^, ^2^ ^]^ The metaverse is a hypothetical platform that provides various immersive experiences with tangible interaction in an expanded virtual space, enabling humans to interact with avatars.^[^ ^3^ ^]^ The metaverse is an expanded concept of previous virtual worlds implemented through extended reality (XR), such as virtual reality (VR), augmented reality (AR), and mixed reality (MR), through continuity and connectivity, which enables exploration of the virtual world through high interoperability. This rapidly evolving technology has permeated various industries, including digital healthcare, where it has the potential to significantly enhance the way healthcare professionals deliver care and interact with patients.^[^ ^4^, ^5^, ^6^, ^7^ ^]^ As a result, metaverse wearables, which incorporate XR and haptic feedback systems, have emerged as promising tools for immersive digital healthcare applications.^[^ ^8^ ^]^

Vivid realization of human senses is a crucial parameter in metaverse technology, and human–machine interface (HMI) and wearable devices can enhance immersion.^[^ ^9^, ^10^, ^11^ ^]^ Most XR systems provide only visual interactions and sounds, which limit realistic immersion feedback. Wearable devices mounted on the human body, including hands, feet, arms, and faces, collect dynamic information such as gestures, eye movements, or the posture of the human body.^[^ ^12^, ^13^, ^14^, ^15^, ^16^, ^17^ ^]^ The signals acquired from the wearables are used to drive an avatar in the metaverse or to provide seamless and natural multimodal sensing such as tactile‐perception or haptic‐feedback.^[^ ^18^, ^19^, ^20^, ^21^ ^]^ Combining prosthetics with XR technology can also provide immersive experiences for rehabilitation to amputees or visually impaired patients.^[^ ^22^, ^23^, ^24^, ^25^ ^]^

Healthcare workers are required to complete high‐risks training, such as procedures of proper disposal handling of biohazardous substances or rapid responses in surgical scenarios.^[^ ^26^, ^27^ ^]^ In addition, the use of telemedicine for diagnosis and prognosis in a virtual world is desirable during pandemics such as COVID‐19.^[^ ^28^, ^29^, ^30^ ^]^ Immersive healthcare solutions can efficiently deliver simulation and guidance of clinical procedure practice, diagnosis, and surgical navigation.^[^ ^31^, ^32^, ^33^ ^]^ Medical procedures can be evaluated in a virtual world under the supervision of experts, and painful responses and emotions can be diagnosed through tactile sensors and haptic feedback.^[^ ^34^, ^35^, ^36^ ^]^ The accuracy of surgical operations can be improved by using AR technology to guide the location of surgical instruments.^[^ ^37^, ^38^ ^]^ Furthermore, AI technology and 5G connectivity facilitate the analysis of acquired healthcare signals more efficiently and solve personalized security issues.^[^ ^39^, ^40^, ^41^ ^]^

**Figure 1:**
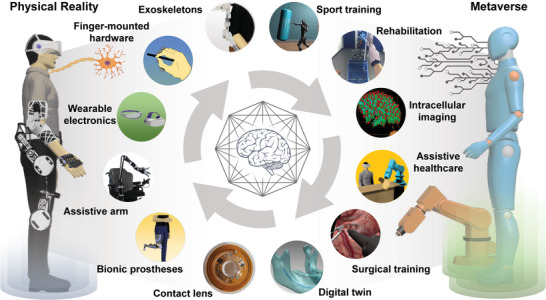
Illustrative schematic of metaverse wearables for immersive digital healthcare. Various tasks such as rehabilitation, intracellular imaging, digital twin, and training are performed in the metaverse through a metaverse‐based human–machine interface (HMI). Sport training: Reproduced with permission.^[^ ^97^ ^]^ Copyright 2021, Nature Portfolio. Rehabilitation: Reproduced with permission.^[^ ^101^ ^]^ Copyright 2021, BioMed Central (BMC). Intracellular imaging: Reproduced with permission.^[^ ^128^ ^]^ Copyright 2022, Frontiers. Surgical training: Reproduced with permission.^[^ ^134^ ^]^ Copyright 2022, Nature Portfolio. Digital twin: Reproduced with permission.^[^ ^151^ ^]^ Copyright 2017, Nature Portfolio. Contact lens: Reproduced with permission.^[^ ^75^ ^]^ Copyright 2020, Nature Portfolio.

This review comprehensively analyzes the current landscape of metaverse wearables and their potential applications in immersive digital healthcare (**Figure** **1**). In recent years, there has been increasing interest in using XR and haptic technologies to facilitate innovative approaches in healthcare. These technologies can improve patient outcomes, increase access to quality care, and enhance practitioner training. Moreover, metaverse wearables can allow healthcare professionals to remotely assess, diagnose, and treat patients, thereby overcoming geographical barriers and addressing healthcare disparities. This review is organized as follows: Initially, we present a comprehensive overview of the fundamental technologies that underlie metaverse wearables, encompassing XR and haptic feedback systems, while discussing their progression and evolution (**Table** **1**). Subsequently, we examine the diverse applications of metaverse wearables within the healthcare sector, including telemedicine, medical twins, rehabilitation, and medical training (**Table** **2**). Following this, we explore the potential advantages and challenges of implementing these technologies in healthcare settings. This article categorizes wearables studies introducing practical demonstrations in virtual worlds or the metaverse. In addition, we included studies in immersive healthcare that were already evaluated through clinical trials and can be of practical help in the medical field. By examining the current state and potential applications of metaverse wearables in healthcare, this review aims to shed light on the transformative potential of this technology and provide guidance for researchers, practitioners, and policymakers in their efforts to leverage the metaverse for improved healthcare outcomes.

**Table 1:** Comparison of the performance of Metaverse wearables.

Mounted location	Sensing/actuation mechanism	Sensing/actuation performance	Accuracy	Application	Reference
Hand	Compliant amplified shape memory alloy actuator	Actuation strain: 300% under 80 g Power density:1.7 kW kg^−1^	Identification rate: 77%	Focus switching	[49]
	Triboelectric tactile sensors/piezoelectric stimulator	Sensing range: ≈0–3.5 N/resonant frequency: 270 Hz	Object recognition: 96%	Surgical training baseball game	[162]
	Linear resonant actuator	Voltage input range: ≈0–11 V	–	Painting, writing, sandbox manipulation	[50]
Skin	Triboelectric nanogenerator (TENG)	Ion (He) dose 1 × 10^16^ ions cm^−2^ (50 keV)	Pattern recognition: 80%	Intelligent, protective suit	[61]
	Piezoresistive thin film/vibratory actuator (Lorentz forces effect)	Relative resistance variation (Δ*R*/*R*0): 0 to 5.92/vibration level ≈20–250 Hz	–	Nursing, biosample collection	[209]
	Resistive sensor	Minimum curvature detection: 0.00014 mm^−1^	Pattern recognition: 98.6%	Electric device remote control	[64]
Arm	Bidirectional triboelectric sensor	DOF: up to 270°/sensing range: over 90 N	–	Sports game	[97]
Foot	Triboelectric sensors	External load resistances from 0.1 to 1000 MΩ	Identification accuracy: 98.4%	Rehabilitation	[87]
	Textile‐based triboelectric sensor	Sensing range: up to 200–300 kPa	Identification accuracy: 96.67%	VR game	[88]
Face	Ball‐typed load cells	Force measurement: smaller than 0.5 N	Pattern recognition: 94%	Food intake detection	[70]
Eye	Electrochromic displays	Potential range: ≈−0.2–0.5 V	–	Navigation	[77]
	Vertical cavity surface emitting laser (VCSEL)	Operating power: 0.5 mW at 850 nm	Angle accuracy: 0.2°	Eye tracking	[75]

**Table 2:** Summary of immersive digital healthcare applications.

Immersive application	Image acquisition approach	Effectiveness	Cost	Programming interface	Refs
Bioimaging	Confocal microscopy	–	$2500	ImageJ, unity	[126]
	Light‐sheet microscopy		–	Unity	[128]
Surgical training	Depth camera	Average 15% score enhancement	Below $4000	Unity, visual studio	[34]
	Laparoscopy	About 80% reduction in surgical time	–	Custom	[133]
Diagnosis	Data acquisition: tactile sensor array	Mean accuracy 83.8%	–	Custom‐written python program	[35]
Surgery guidance	Endoscopic camera	–	–	Da Vinci surgical system	[210]
	Head‐mounted display camera, computed tomography (CT) scan	Error across all patients: 1.442 ± 0.234 mm	$3500	Geomagic Control X	[32]

## 2 Metaverse Wearables Coupled with XR Technology

Interactive wearable devices that detect and stimulate various signals of body parts are required to connect the physical world and the metaverse seamlessly. Wearables linked with XR have the potential to be applied to the immersive healthcare applications of the metaverse by transmitting interaction signals. This chapter introduces various studies of wearable devices that can interact with diverse signals in the physical world by attaching to various parts of the body and have the potential for applications in immersive healthcare.

### 2.1 Wearable Haptic Devices for Arms and Hands

**Figure 2:**
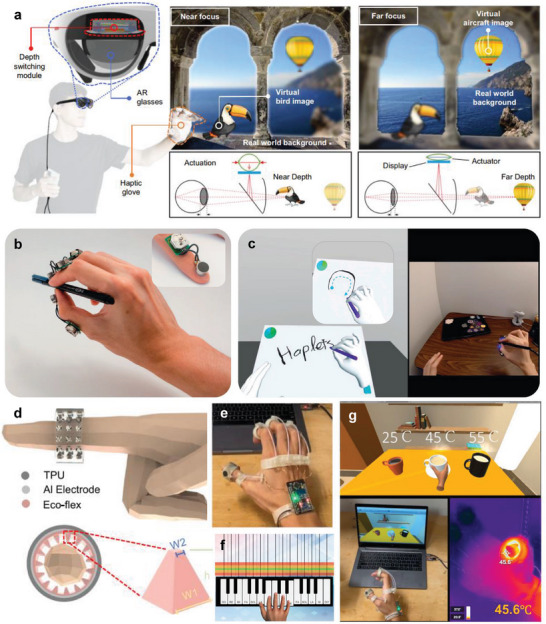
Wearable haptic sensors. a) A haptic glove controls depth‐variable AR glasses with a shape memory alloy actuator. Reproduced with permission.^[^ ^49^ ^]^ Copyright 2022, Nature Portfolio. b) In VR and AR, a finger‐worn haptic device for hand tracking and haptic feedback applications. c) Rendering output of drawing through the finger‐worn haptic device. Reproduced with permission.^[^ ^50^ ^]^ Copyright 2021, Frontiers. d) Multimodal haptic feedback rings with triboelectric and pyroelectric sensors for immersive interactions. e) A photograph of the multimodal sensing platform. Demonstration of f) playing virtual piano and g) temperature feedback in the virtual space. Reproduced with permission.^[^ ^51^ ^]^ Copyright 2022, Nature Portfolio.

Hands and fingers provide a highly developed sense of touch and haptic feedback, which allows one to perceive and interpret textures, shapes, and temperatures.^[^ ^42^, ^43^, ^44^ ^]^ In addition, various motions of the hands, such as picking up objects or using a smartphone, are required in daily life. Thus hand‐worn advanced haptic devices have been developed for more sophisticated cognition and feedback.^[^ ^45^, ^46^, ^47^, ^48^ ^]^ Multi‐focus AR glasses with an artificial muscle actuator were introduced, driven by adjusting focus through a soft haptic glove (**Figure** **2**a).^[^ ^49^ ^]^ A shape memory alloy (SMA) actuator integrated with the AR glasses controls the position of the display to adjust the focusing distance between the human eye and the virtual objects. The SMA actuator has a bistable parallelogram linear stage structure to overcome the inherent limitations of SMA, such as nonlinear mechanical properties, and to reduce undesirable motions, such as tilting and rotation. An SMA is also incorporated in the fingertip parts of a soft haptic glove, which was determined to detect external force as a resistance‐type pressure sensor. Appropriate image depth for users can be manually selected through the actuation and pressure sensing of SMAs. This approach will help increase the stereoscopic reality of object distances in the metaverse and adjust the device according to individual eyesight. A finger‐mounted haptic device, *Haplets*, was developed for XR applications (Figure 2b).^[^ ^50^ ^]^ The Haplets include a linear resonant actuator (LRA), integrated circuit boards, and a battery for vibrotactile stimulus.

The LRA offers pressure and texture feedback to the finger through vibration; thus, the Haplets can emulate sensations when holding an object or rubbing the surface of an object in a virtual platform. The Haplets also provide immersive experiences to the user by implementing vibration motions for various actions, such as drawing with a pen or hammering and spraying (Figure 2c). Recently, a multimodal sensing and feedback platform was introduced for thermo‐tactile sensing and thermo‐haptic feedback (Figure 2d,e).^[^ ^51^ ^]^ The ring‐shaped platform perceives pressure and temperature through triboelectric/pyroelectric sensors and offers the feedback of vibration and heat using vibrators and a nichrome heater. The multimodal ring can render the feeling of holding hot coffee in the metaverse world and provide the immersive experience of playing the piano through vibration (Figure 2f,g). The platform implements the motions of a robot by emulating hand motions and recognizes various finger motions with high accuracy. The wearable haptic devices can be applied to immersive surgical training or robot‐assisted telesurgery applications.

### 2.2 Skin Electronics for Multimodal Feedback

**Figure 3:**
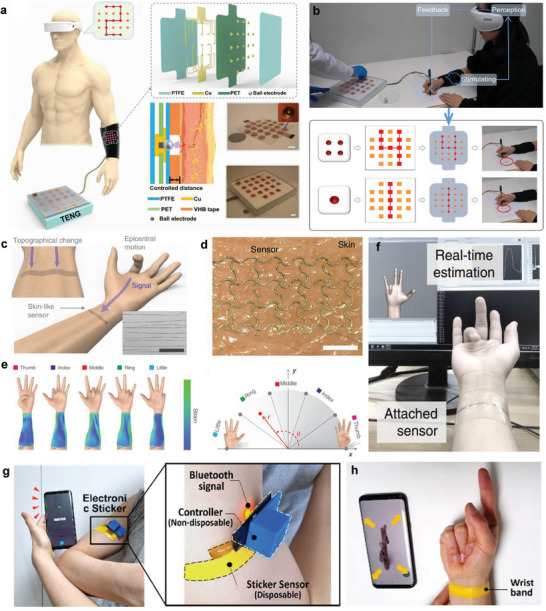
Wearable electronic skin sensors. a) Diagram of an electro‐tactile (ET) system for realizing virtual tactile experiences. b) Demonstration of tactile VR experiences by electrical stimulation of TENG array. Reproduced with permission.^[^ ^61^ ^]^ Copyright 2021, American Association for the Advancement of Science (AAAS). c) Schematics of ultrasensitive skin electronics for detecting epicentral motions. d) A photograph of a motion detection sensor attached to the skin. e) 3D color map of skin deformation according to finger folding. f) Rendering of virtual hand motion. Reproduced with permission.^[^ ^62^ ^]^ Copyright 2020, Nature Portfolio. g) Photographs of skin‐interactive wearable sensors for recording skin wave fluctuations. h) Remote control by a finger gesture. Reproduced with permission.^[^ ^64^ ^]^ Copyright 2022, MDPI.

Skin performs various sensory functions, including temperature, pressure, and vibration, and delivers several biomedical signals, such as electromyography (EMG) and electrooculography (EOG).^[^ ^52^, ^53^, ^54^ ^]^ Skin‐like electronics, which can be integrated with human skin seamlessly, emulate the properties of skin for healthcare applications.^[^ ^55^, ^56^, ^57^, ^58^, ^59^, ^60^ ^]^ An electro‐tactile (ET) system that electrically stimulates skin has been developed for virtual tactile experiences (**Figure** **3**a).^[^ ^61^ ^]^ The ET device features a triboelectric nanogenerator (TENG) for controlling electrostatic discharge stimulation and an ET interface with ball electrodes for electrostimulation to the skin. Charges are generated by the electrostatic induction effect when a pattern is input to the TENG. A potential difference between the ball electrode and the skin is promoted due to charge accumulation, which induces instantaneous current flow to the skin by a discharge. Electrical stimulation provides participants with a tactile VR experience, recognizing patterns of letters or figures and enhancing immersive interactions through a VR headset (Figure 3b). Not only visual information, but tactile interaction can improve the immersive experience. Hence, virtual tactile communications can use the system for tactile prosthetics feedback. A motion detection sensor was developed for monitoring finger movement using ultrasensitive skin electronics attached to the wrist (Figure 3c).^[^ ^62^ ^]^ The sensor, fabricated through laser‐induced nanoscale cracking, offers ultra‐sensitive detection of small skin deformations (Figure 3d). The detection signals were classified according to finger motion through a deep neural network, and the complex motion of five fingers was projected on a virtual platform in a real‐time (Figure 3e,f). In a similar method, a device has been introduced to control a robot by detecting the movement of the muscles on the wrist through a skin‐like sensor.^[^ ^63^ ^]^ This skin‐like sensor is fabricated through additive nanomanufacturing for biocompatibility and anti‐oxidation, providing high precision.

Similarly, a thin electronic sticker made of a pressure‐sensitive material has also been introduced for detecting body motions through epidermis deformation (Figure 3g).^[^ ^64^ ^]^ Furthermore, the sensor can remotely control a personalized device, such as a smartphone, according to body movements (Figure 3h). The approaches predicting finger movement through wrist skin deformation can reduce the heterogeneity of remote therapy by alleviating the hand limitations by more than the previous wearable haptic devices integrated into the hands.

A flexible EOG sensor and a VR system were developed for eye vergence detection and therapies.^[^ ^65^ ^]^ EOG signals can be observed according to the convergence and divergence of ocular motions when the EOG sensor is attached to the face, and the signals are classified through signal processing to optimize vergence analysis. In addition, the device provides training for eye vergence treatment through images provided by a VR headset, and the feedback of the EOG sensor can increase treatment efficiency. An approach for obtaining information on the voices of patients who have lost their voice has been introduced using an epidermal sEMG patch.^[^ ^66^ ^]^ The voice information can be recognized at the jaw and face, and the patch collects the sEMG signals for silent speech recognition. Several instructions for silent speech recognition were classified by decomposition and pattern recognition, and the instructions interacted with virtual characters representing emotions.

### 2.3 Eyeglasses and Contact Lens‐Type Wearable Devices

Glasses and contact lenses are commonly used wearable tools. Smart glasses, including displays, provide superimposed information augmenting the vision,^[^ ^67^, ^68^ ^]^ and electronic contact lenses can be used to diagnose body fluid.^[^ ^67^, ^68^, ^69^ ^]^ Glasses and contact lens‐type wearable devices can measure facial muscle movements and eye tracking besides XR applications.

**Figure 4:**
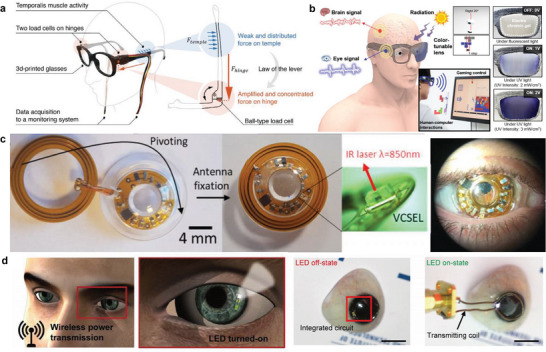
Glasses and contact lens‐type wearables. a) A schematic illustration of GlasSense measuring force signals of the temporalis muscle activity through ball load cells. Reproduced with permission.^[^ ^70^ ^]^ Copyright 2017, Nature Portfolio. b) Electronic glasses for monitoring biological signals and performing assigned roles. Reproduced with permission.^[^ ^71^ ^]^ Copyright 2020, American Chemical Society. c) A contact lens combined with near‐infrared illumination for eye tracking. Reproduced with permission.^[^ ^75^ ^]^ Copyright 2020, Nature Portfolio. d) Smart contact lenses with light‐emitting diode (LED) for glucose monitoring wirelessly. Reproduced with permission.^[^ ^76^ ^]^ Copyright 2018, AAAS.

A glass‐type wearable device, GlasSense, was developed to observe the temporalis muscle's minuscule vibration patterns (**Figure** **4**a).^[^ ^70^ ^]^ Two ball load cells are located on the hinge of 3D‐printed glasses, detecting the minute force of the temporalis muscle and converting the force into electrical signals. The GlasSense system can accurately classify behaviors such as chewing, talking, and winking through a support vector machine algorithm. After acquiring facial movements using the wearable glasses, emotions or facial expressions can be translated to the metaverse world avatar.

Electronic glasses have been introduced to acquire signals such as EOG and electroencephalogram (EEG), respond to ultraviolet (UV) signals, and detect body movements (Figure 4b).^[^ ^71^ ^]^ The glasses were equipped with EEG, EOG, UV sensors, and accelerators, and the transmittance of the glasses can be controlled according to UV intensity through an indium tin oxide (ITO)‐coated polyethylene terephthalate (PET) film. The EEG signal detects the brain activity signals, and the EOG signal observes the movement of the eyes. The acquired electrophysiological signal from the sensors can transmit commands by connecting to a computer interface.

Contact lenses with integrated illumination components and electronic circuits have recently been introduced for various applications.^[^ ^72^, ^73^, ^74^ ^]^ A contact lens with a near‐infrared (IR) VCSEL laser pointer has been developed for eye tracking (Figure 4c).^[^ ^75^ ^]^ The laser pointer beam on the surface of a beam splitter can be monitored through an IR camera to analyze the direction of the line of gaze.

The smart contact lens illumination can also be used for real‐time glucose monitoring (Figure 4d).^[^ ^76^ ^]^ The contact lens controls the operation of display pixels by the relative change in resistance according to glucose concentration. An electrochromic (EC) display contact lens using Prussian blue micro‐patterning can visualize the direction of movement through visual augmentation for navigation applications.^[^ ^77^ ^]^ The contact lens is a wearable device that can potentially replace VR headsets, although there are currently limitations in implementation due to low‐resolution and clinical risk. A smart augmented contact lens with low heat generation and sufficient visual resolution is required to ensure safety and enhance immersion.

### 2.4 Wearable Foot Electronics for Gait Analysis and Training

**Figure 5:**
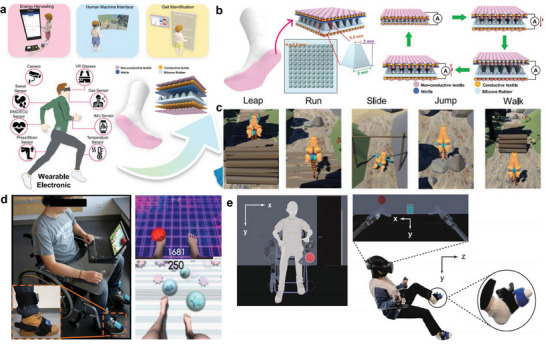
Wearable foot electronics for gait analysis and training. a) Schematic illustrations of wearable triboelectric sensory systems for detecting gait and waist motions. b) The configuration and working principle of triboelectric nanogenerator (TENG) sensors. c) Real‐time virtual emulation of diverse motions, including running, walking, and jumping. Reproduced with permission.^[^ ^88^ ^]^ Copyright 2020, Nature Portfolio. d) A home‐based training platform for rehabilitation of lower limb muscles. Reproduced with permission.^[^ ^89^ ^]^ Copyright 2017, Frontiers. e) An interaction with a vibro‐transducer during the virtual tasks using robotic limbs. Reproduced with permission.^[^ ^90^ ^]^ Copyright 2022, Nature Portfolio.

Gait analysis is commonly used to diagnose behavioral diseases or improve athletic ability by analyzing personal walking or running patterns.^[^ ^78^, ^79^, ^80^ ^]^ Gait analysis is quantified by the foot pressure or the motion of the ankle or knee with various devices, including wearable sensors or cameras.^[^ ^81^, ^82^, ^83^ ^]^ In particular, wearable sensors can increase the efficiency of rehabilitation through long‐time monitoring.^[^ ^84^, ^85^ ^]^ A capacitive pressure sensor (CPS) array was introduced for real‐time monitoring of static and dynamic foot pressure.^[^ ^86^ ^]^ The device consists of a dielectric layer fabricated by laser cutting between the CPS array and a bottom electrode. The pore size in the dielectric layer is optimized according to the capacitance. The pressure signals were transmitted through wireless communication and visualized foot pressure mapping according to postures. A triboelectric sensory system has been developed for waist motion and gait analysis (**Figure** **5**a).^[^ ^87^ ^]^ The sensor contains pyramid‐shaped triboelectric layers, which generate voltages to detect pressure signals (Figure 5b). The triboelectric sensor was installed in a belt to measure the waist's rotation direction, and the foot's pressure patterns were analyzed through sensors installed in a shoe. The system learned the acquired unique gait data for patient identification. It was applied to a gait aid robot system for rehabilitation. Similarly, smart socks containing triboelectric sensors also introduced an approach of emulating motions, such as running, walking, and jumping with a virtual character (Figure 5c).^[^ ^88^ ^]^

A home‐based training platform has been introduced for rehabilitating spinal cord injury patients through wearable accelerometers (Figure 5d).^[^ ^89^ ^]^ The platform collects foot movement signals by attaching three‐degree‐of‐freedom accelerometers to the dorsum of the foot and the tibias. The foot movement signals were transmitted to an avatar through Unity, allowing the avatar to perform tasks such as juggling footbags or kicking balls in the virtual space. An approach has also been developed to stimulate the feet through wearable transducers for perceiving signals from the virtual world (Figure 5e).^[^ ^90^ ^]^ While performing the crossmodal congruency task through robotic limbs in VR, participants recognize the visuotactile stimulus feedback when the robot arm touches a specific object. The developed foot electronics perform only sensing or stimulation functions; a wearable device that simultaneously acquires stimulation and motion signals is required to increase the immersive experience further.

### 2.5 Exoskeleton and Prosthesis for Rehabilitation as Assistive Healthcare Tools

**Figure 6:**
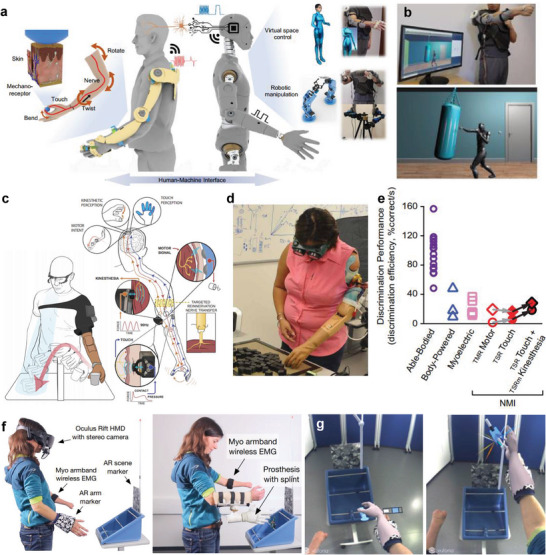
Exoskeleton and prosthesis for virtual rehabilitation training. a) Schematic illustrations of an exoskeleton system to realize corresponding motions in a virtual or robotic world. b) The implementation of boxing training through the exoskeleton. Reproduced with permission.^[^ ^97^ ^]^ Copyright 2021, Nature Portfolio. c) A bionic upper limb provides a bidirectional connection between the patients and the prosthetics. d) The demonstration of prosthesis efficiency and profitability (PEP) assessment. e) Measured discrimination performance results. Reproduced with permission.^[^ ^98^ ^]^ Copyright 2021, AAAS. f) Experimental setup for immersive AR system to visualize a virtual hand. g) Participant perspective on the virtual hand and objects. Reproduced with permission.^[^ ^101^ ^]^ Copyright 2021, BMC.

Physical rehabilitation is crucial for patients with musculoskeletal disorders or limb amputations to restore their lost function, increase their range of motion, and alleviate pain.^[^ ^91^, ^92^, ^93^ ^]^ In addition, exoskeletons and prostheses linked with the XR platforms can increase rehabilitation efficiency and enable remote training.^[^ ^94^, ^95^, ^96^ ^]^ A customized exoskeleton with cost‐effective fabrication and low power consumption has been developed to detect and display the joint movements of users (**Figure** **6**a).^[^ ^97^ ^]^ The triboelectric bidirectional sensors embedded in the exoskeleton monitor multidimensional motions such as rotation, twisting, and linear motion. The 3D‐printed exoskeleton is mounted on the arm, shoulder, and hand and receives triboelectric signals from the sensors for virtual object motions. The exoskeleton system is linked with an avatar in the virtual space, which implements various locomotions such as ping‐pong and punching (Figure 6b). Interoperability through the avatar can be expanded to various applications, including rehabilitation in the metaverse.

A bionic prosthetic limb that detects neural signals and connects with robots has been introduced to replace the body functions of patients with limb amputation (Figure 6c).^[^ ^98^ ^]^ Haptic perception and motor intentions are detected through targeted muscle and sensory reinnervation for the intended movement of patients (Figure 6d,e). The motor performance and sensory discrimination were quantified while the participants wore translucent glasses and headphones to ensure experimental accuracy. Similarly, a device that provides a virtual haptic representation has also been developed by attaching a flexible electronic sensor to the epidermis near the prosthesis.^[^ ^22^ ^]^ The principle of haptic perception and motor intentions that predict patients' intended movement also can be applied to manipulate avatars or virtual objects in the hypothetical platform.

Prosthetic controls require considerable effort and training, but XR devices can offer engagement and motivation for rehabilitation through an immersive experience.^[^ ^99^, ^100^ ^]^ An immersive AR system visualizing a virtual hand on an amputated area was introduced for virtual training (Figure 6f).^[^ ^101^ ^]^ The intended movement and grasping force patterns are recognized from the EMG sensor and command the prosthesis to move. The corresponding feedback is displayed as an overlay in a first‐person perspective when participants wear a head‐mounted display (Figure 6g). Such prosthetic training in the virtual space can provide confidence to patients to perform tasks in the metaverse and the physical world.

### 2.6 Implantable Visual Prosthesis

Patients with visual impairment or retinal degenerative diseases need a device that complements the visual sensor organ.^[^ ^102^, ^103^ ^]^ For patients who have lost the function of photoreceptors, a sub‐retinal implant device with a photovoltaic pixel array is used to convert light into electrical signals and stimulate neurons.^[^ ^104^ ^]^

**Figure 7:**
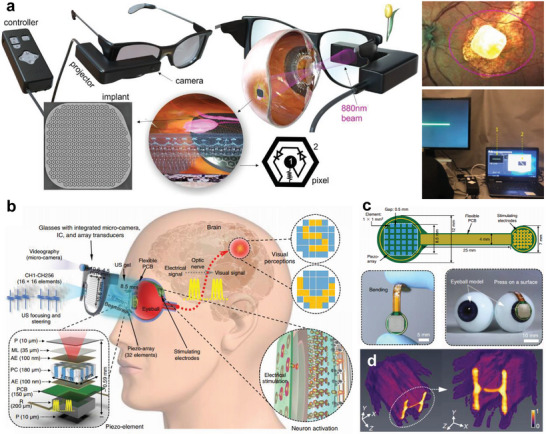
Implantable visual prosthesis. a) A PRIMA system with AR glasses for age‐related macular degeneration (AMD) patients. The visual information acquired by a micro camera is transmitted to an artificial photovoltaic pixel array through a NIR projector. Reproduced with permission.^[^ ^109^ ^]^ Copyright 2022, Nature Portfolio. b) Diagram of ultrasound‐induced visual prosthesis a 2D array transducer. c) The design and photographs of 2D piezo‐array. d) Enhanced simulated intensity emitted from a 2D array transducer. Reproduced with permission.^[^ ^111^ ^]^ Copyright 2022, Nature Portfolio.

However, the previous visual prostheses have side effects such as photophobic and phototoxic effects under bright illumination and are limited by low‐resolution due to the small number of photovoltaic pixels.^[^ ^105^ ^]^ 880 nm near‐infrared light is used to prevent the photophobic and phototoxic effects, and visual information is transmitted to photovoltaic electrodes through wearable glasses with a microdisplay.^[^ ^106^ ^]^ Several devices have been approved by the US Food and Drug Administration (FDA), but the FDA has only approved the use of visual prostheses for the almost entirely blind. Argus I/II model has been demonstrated in retinitis pigmentosa (RP) patients,^[^ ^107^, ^108^ ^]^ and a PRIMA system has been applied for age‐related macular degeneration (AMD) patients (**Figure** **7**a).^[^ ^109^, ^110^ ^]^

An implantable eye was developed by implementing visual effects through ultrasound stimulation (Figure 7b).^[^ ^111^ ^]^ A piezo‐array is attached to the eyeball pupil and detects ultrasound stimulation transmitted by a 2D array transducer in wearable glasses (Figure 7c). Images are acquired through a micro‐camera and converted to digital data. The signal is then processed by a Verasonics system, that is, 2D array transducer driver, and is transmitted through ultrasonic focusing and steering (Figure 7d). The stimulated ultrasound signal is transformed into an electrical signal and transmitted to the array electrode, which is attached to the retinal surface through the flexible PCB. High‐density electrodes are required to achieve the highly immersive vision of an implantable visual prosthesis. A high‐density and curved sensor array similar to the photoreceptor of the human eye is being developed to provide visual disorder patients with realistic experiences.^[^ ^112^, ^113^, ^114^ ^]^ Materials such as molybdenum disulfide (MoS_2_) and graphene are promising candidates for curved image sensor arrays because of their superior absorption, photoresponsivity, and fracture strain characteristics.^[^ ^115^, ^116^ ^]^ However, Implantable visual prostheses still have limitations in providing insufficient experiences of the metaverse due to low‐resolution. Along with the development of visual prosthetic systems, the progress of an alternative metaverse platform is required to offer an experience of the virtual world, even at low resolution, to the visually impaired.

## 3 Immersive Digital Healthcare Applications

Not only evolved wearable devices, but advanced visually augmented platforms can provide immersive experiences in digital healthcare applications. Immersive digital healthcare has been expanded to various fields, such as biomedical imaging, training, surgery, and immersive technology, which can help improve therapeutic effects or surgical performances. This section introduces diverse digital healthcare applications that can transcend activities in the physical world through visual augmentation.

### 3.1 Interactive Visualization for Bio/Medical Applications

3D volumetric microscopic imaging is a crucial technique for investigating cell functions and architectures for biomedical applications.^[^ ^117^, ^118^, ^119^ ^]^ However, microscopic images are commonly observed on a 2D display, which cannot express complex spatiotemporal bio‐structures.^[^ ^120^, ^121^ ^]^ New XR visualization approaches can allow 3D exploration of intracellular architectures.^[^ ^122^, ^123^, ^124^ ^]^ The approaches can more accurately analyze multidimensional data by selecting and annotating subregions.^[^ ^125^ ^]^

**Figure 8:**
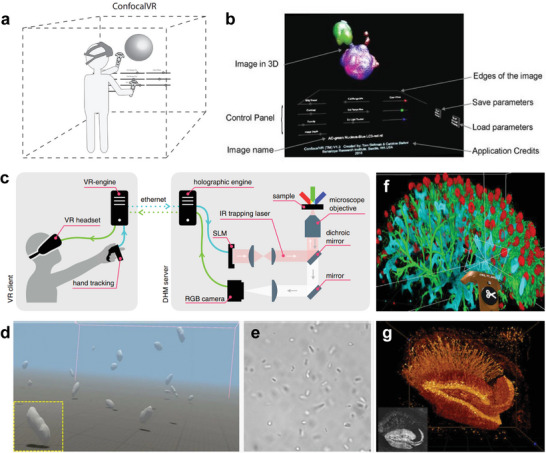
Interactive visualization for biomedical applications. a) A schematic illustration of a confocal VR system to detect confocal stacks. b) A user interface for adjusting visual parameters. Reproduced with permission.^[^ ^126^ ^]^ Copyright 2018, Elsevier. c) Diagram of immersive manipulation through VR platform and holographic microscopy. d) Real‐time 3D rendering and e) bright field imaging of swimming *E. coli* bacteria. Reproduced with permission.^[^ ^127^ ^]^ Copyright 2021, Nature Portfolio. f) VR interface with cloud computing for image annotation. g) A rendering image of mouse hippocampus captured by two‐photon microscopy. Reproduced with permission.^[^ ^128^ ^]^ Copyright 2022, Frontiers.

A confocal VR system was developed to observe the image stack of cellular structures through a VR headset (**Figure** **8**a).^[^ ^126^ ^]^ Hand controllers can adjust visual parameters such as the brightness and contrast of a rendered 3D image and rotate and scale the image (Figure 8b). The device allows several scientists to observe 3D volume images simultaneously and communicate through a microphone mounted on a headset.

A novel VR interface has been introduced for real‐time immersive manipulation using holographic microscopy.^[^ ^127^ ^]^ Bacteria or silica microspheres can be controlled by modulating 3D multi‐traps through holographic optical tweezers. The holographic engine, connected with the VR engine, controls a spatial light modulator (SLM) that creates focal spots in the 3D volume for independent optical traps (Figure 8c). The VR engine, linked with the VR headset, generates an immersive visualization of swimming bacteria through volumetric reconstruction with real‐time rendering (Figure 8d,e).

An approach merging VR with cloud computing was introduced for biological data annotation and analysis (Figure 8f).^[^ ^128^ ^]^ An annotating step is crucial for training machine learning algorithms or visualization of data analysis. VR tagging with the VR controller and headset allows the acceleration of image annotation by observing 3D data more efficiently. The approach can be applied to various microscopy modalities, such as light‐sheet microscopy or two‐photon imaging for analyzing biological structures (Figure 8g). Previous studies demonstrated virtual visualization through microscopic imaging acquired in‐vitro, but XR visualization acquired from in‐vivo imaging will allow efficient real‐time optical biopsy diagnosis.

### 3.2 Metaverse Platforms with XR for Medical and Nursing Education

The cutting‐edge technology of the metaverse holds practical applications not only in medical and nursing education, but also in team training for healthcare professionals in the clinical field. For instance, medical and nursing simulation training can be conducted using metaverse wearables. By leveraging avatar technology, we can offer simulations that interact with virtual patients and healthcare workers within a virtual environment. Through these experiences, students can enhance their clinical skills by monitoring the physical state of patients within the virtual realm and executing medical or nursing procedures via wearable devices.

**Figure 9:**
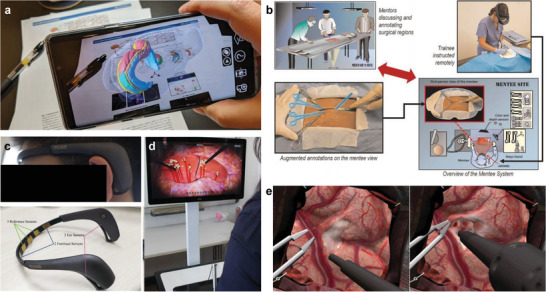
Immersive medical education applications. a) A platform that augments 2D literature data with 3D AR information. 3D complex information can be observed through a smartphone or a web‐based viewer. Reproduced with permission.^[^ ^132^ ^]^ Copyright 2022, Nature Portfolio. b) Diagram of telementoring platform for surgical procedure training. Augmented surgical procedures annotated by a mentor are projected from the mentee's perspective. Reproduced with permission.^[^ ^34^ ^]^ Copyright 2020, Nature Portfolio. c) A VR training system with a wearable ECG sensor. d) Demonstration of VR laparoscopic surgery simulation. Reproduced with permission.^[^ ^133^ ^]^ Copyright 2022, BMC. e) VR simulation of ultrasonic aspiration through a continuous expertise monitoring system. Reproduced with permission.^[^ ^134^ ^]^ Copyright 2022, Nature Portfolio.

Studies in which medical training with surgical scenarios is provided through avatars in the metaverse with XR environments have been conducted in various medical fields such as radiology, internal medicine, nursing, and neurology.^[^ ^1^, ^129^, ^130^, ^131^ ^]^ The experiences allow medical students to enhance medical technology skills in a risk‐free decision‐making environment by learning surgical procedures and reviewing personalized feedback. A technology that visualizes digital data through AR and web‐based viewing has been developed to offer immersive information in research publications (**Figure** **9**a).^[^ ^132^ ^]^ Previous scientific output, such as an article, has limits in providing effective information because the output confines complex data to 2D static figures. The developed framework provides 3D AR information visualization through personalized devices such as smartphones or a web‐based‐viewer to augment science communication digital media and reduce the gap between the 2D data in the article and the actual model.

A telementoring platform that provides augmented visualization of remote guides was introduced to increase proficiency in surgical procedures (Figure 9b).^[^ ^34^ ^]^ A remote professional surgeon guided operation sequences and arrangement of surgical instruments to conduct training in strict cricothyroidotomy surgical procedures, and training participants were assessed for completion time and surgical performance. The overall surgical understanding was scored with various quantitative indicators such as emergency cricothyroidotomy performance scores, global rating scale, and completion time. As a result, the telementoring platform achieved higher scores than the audio guide.

A quantitative analysis approach was also introduced to evaluate the VR training performance of surgical instruments such as laparoscopy.^[^ ^133^ ^]^ Fifty‐one participants wearing an ECG sensor were trained for four tasks through a VR laparoscopic surgery simulator (Figure 9c). Most of the participants scored higher on the index of performance ability after training than before training, and the average heart rates of participants also decreased after training (Figure 9d). The results mean that VR training can help participants improve surgical performance skills and reduce their cognitive load.

Similarly, a VR surgical simulation platform that evaluates proficiency through deep learning has been developed (Figure 9e).^[^ ^134^ ^]^ The platform continuously monitors and learns the trainee's knowledge of surgical technical skills through performance metrics such as movement, pressure, and bimanual skills. The approach can provide appropriate training stages according to the skill levels of trainees. The previous XR for medical training has been conducted through already‐designed virtual environments. However, a new approach to medical training models the surgical conditions of personalized patients through medical images such as endoscopy or computerized tomography (CT).

Last, applying simulation education that replicates hard‐to‐experience scenarios in the clinical field, such as flood disasters and wartime situations, can be exceptionally effective.^[^ ^135^, ^136^ ^]^ In light of recent devastating disaster events like floods and earthquakes, enhancing the realism in disaster nursing and medical simulation training could be significantly beneficial. The application of simulation training, specifically tailored to address the treatment of patients in international warfare situations, could prove highly effective. This strategy fosters preparedness for extreme circumstances and cultivates the capacity for rapid and effective responses to various emergency scenarios in the healthcare field.

### 3.3 Assistive Technology for Immersive Digital Healthcare

Assistive technology or assistive healthcare is a rehabilitative technology that minimizes reliance on medical care and long‐term care, ultimately helping to combat exclusion related to disability or age.^[^ ^137^ ^]^ Metaverse wearable platforms can help patients maximize the effects of rehabilitation by enabling tasks that are never experienced in the real world.^[^ ^138^, ^139^ ^]^ In addition, avatar‐based rehabilitation can reduce the heterogeneity of the virtual environment to patients through immediate feedback.

A novel metaverse interface was introduced through avatars assisted by a robot arm for physical manipulation tasks.^[^ ^140^ ^]^ The robot arm replicates the user motion acquired through a camera, and the avatar motions visualize through a head mount display for visual illusion. The action of pouring water with the robot acting as an intervention was demonstrated through the metaverse interface, and the error between the virtual hands and the intervention robot was analyzed.

A mirror therapy approach with VR for upper limb recovery has been introduced to reduce the sense of heterogeneity by patients.^[^ ^141^ ^]^ This approach offers the illusion of arm motions in the hemiparetic upper limb through an avatar, and the usability of patients was verified through evaluation methods, including the Fugl‐Meyer Upper Extremity and the Action Research Arm. Demonstrating mirror therapy through the avatar can be significantly encouraging in securing connectivity and interoperability in other applications for the metaverse. Although the evaluation results have a limitation in achieving statistical significance, this method can be extended to various applications for treatment purposes in virtual platforms.

In summary, metaverse wearables hold significant potential as an assistive healthcare tool, effectively applied in the treatment and education of rehabilitative patients. For instance, such patients can participate in strength enhancement exercises or balance training within an avatar‐based virtual environment linked to a robotic arm. This technology‐enabled approach facilitates patient‐led therapy, fostering increased independence and improved functionality throughout rehabilitation. Furthermore, the use of metaverse technology extends to the surgical arena within the healthcare field. This demonstrates the far‐reaching applications of this advanced technology and its immense potential in digital twin and digital medicine.

### 3.4 Digital Twins for Diagnostics and Therapeutics

A digital twin is a virtual representation technique that imitates and updates target systems' physical structure, context, and behavior in real‐time for simulation, monitoring, or maintenance purposes.^[^ ^142^, ^143^, ^144^, ^145^ ^]^ Digital twins are mainly targeted at engineering systems but can also be applied to various fields, such as medical and information systems that perform critical decisions in surgical or physical systems.^[^ ^146^, ^147^ ^]^ Digital twins can efficiently derive diagnoses and prognoses for each patient and precisely adjust health recovery through computational modeling predictions.^[^ ^148^, ^149^, ^150^ ^]^

**Figure 10:**
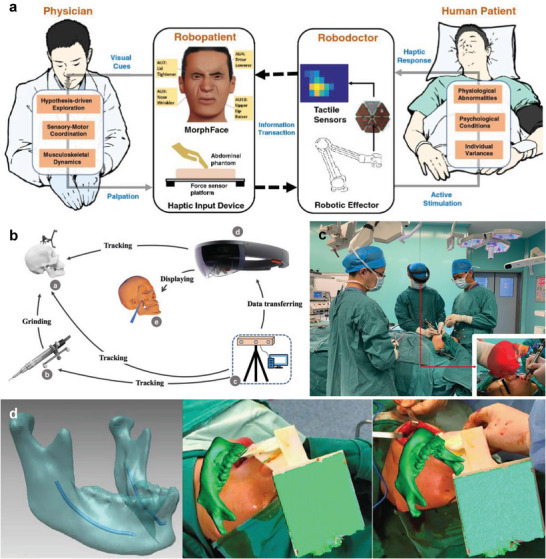
Digital twins for diagnosis and surgical treatment. a) Schematic illustrations of a face‐mediated human–robot interaction system. The haptic response is measured through tactile sensors, and the measured force is rendered as a facial expression. Reproduced with permission.^[^ ^35^ ^]^ Copyright 2022, Nature Portfolio. b) Diagram of an AR surgical navigation system. c) Clinical operation image by the surgeon. Reproduced with permission.^[^ ^37^ ^]^ Copyright 2021, Nature Portfolio. d) Digital twin rendering for intraoperative guidance. Reproduced with permission.^[^ ^151^ ^]^ Copyright 2017, Nature Portfolio.

A face‐mediated, human‐robot interaction was developed to provide visual feedback in telemedicine (**Figure** **10**a).^[^ ^35^ ^]^ The tactile sensor array measures the tactile response of a patient during diagnosis and transmits the acquired data to the robot to render the palpation force as a facial expression. The strength and spatial extent of palpation can be analyzed by visualizing the degree of pressure on a tactile map, and the rendered facial expression changes depending on the amount of pressure. The face‐mediated human‐robot interaction approach provides intuitive palpation feedback for remote diagnosis without risk to participants.

An AR navigation system has been introduced to increase the surgical efficiency and safety of craniofacial fibrous dysplasia (Figure 10b).^[^ ^37^, ^151^ ^]^ Symmetrical reconstruction of a face is required to treat fibrous dysplasia, and the treatment should minimize position errors of surgical instruments such as drills for high surgical success. The system tracks the location of the drill, and the virtual modeling is rendered in green so if the drill is far (>1 mm) from the target surface and in red if the target is close (≤1 mm) (Figure 10c,d). The navigation system allows surgeons to intuitively judge the reconstruction boundary and depth with a resolution of 1.442 ± 0.234 mm. A study was also conducted to evaluate the safety and feasibility of urological surgery by augmenting medical images such as X‐ray and CT scans with smart glasses, and the approaches of augmenting surgical information will potentially improve surgical performances in various fields.^[^ ^152^ ^]^

### 3.5 AI and Machine Learning in Immersive Digital Healthcare

AI can perform tasks such as visual recognition, reasoning, and learning that were previously performed by human intelligence, and machine learning is a process of learning data for efficient pattern recognition.^[^ ^153^, ^154^, ^155^, ^156^ ^]^ AI and machine learning technologies can improve the realistic interactions between humans and avatars in the metaverse, and the expansion of AI‐based metaverse to the healthcare field can significantly impact clinical practice and human health.^[^ ^157^ ^]^ For example, information obtained through medical equipment or HMI devices can be shared through a cloud system, and learned medical information could provide patient feedback through AI‐based analysis tools.^[^ ^158^, ^159^, ^160^ ^]^

**Figure 11:**
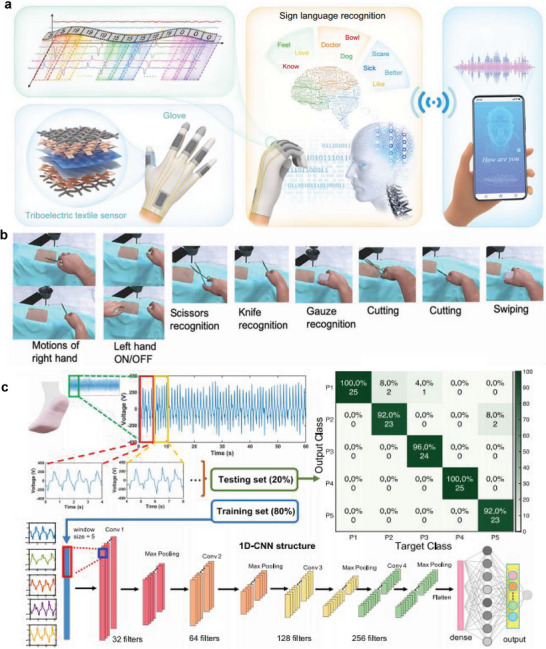
Artificial intelligence for immersive healthcare. a) Schematic illustrations of an AI‐based smart glove system with triboelectric sensor for sign language recognition and communication. Reproduced with permission.^[^ ^161^ ^]^ Copyright 2021, Nature Portfolio. b) VR surgical training program uses the finger motion recognition of the haptic glove. Reproduced with permission.^[^ ^162^ ^]^ Copyright 2020, AAAS. c) Gait analysis through deep‐learning‐based smart sock signals. The CNN model analyzes and predicts the gait recognition of participants through the acquired data. Reproduced with permission.^[^ ^88^ ^]^ Copyright 2020, Nature Portfolio.

An AI‐based smart glove system has been developed for sign language recognition and communication (**Figure** **11**a).^[^ ^161^ ^]^ The glove has a triboelectric sensor that detects motion signals, and acquired data are classified into words. The system analyzes sign‐sensing signals for data comprehension and uses non‐segmented and segmented AI framework frames for word and sentence recognition. The AI‐based system can distinguish 50 words and 20 sentences, with an accuracy of 91.3% and 95%, respectively. Similarly, a technique capable of performing VR surgical training also has been developed for performing the motion of surgical operation scenario gestures in a virtual space through an AI‐based haptic glove (Figure 11b).^[^ ^162^ ^]^

An approach to analyzing data acquired from smart socks through AI technology was developed for efficient gait analysis.^[^ ^88^ ^]^ The CNN‐based method was selected because the algorithm can analyze human activity data efficiently, and a model to distinguish participants was designed through AI‐based gait analysis. Parameter data such as amplitude, period, and gate cycle repeatability were learned, and the network system consisting of convolutional layers, max‐pooling, and connected layers predicted the output of participants. The model classified 13 participants with an accuracy of about 93.54% and five participants with an accuracy of 96.67%. These AI‐based cognitive technologies can be extended to offer personalized training directions. The recognition algorithms will help to increase the efficiency of remote rehabilitation by providing the appropriate rehabilitation intensity and time for each individual.

### 3.6 Internet of Things and 5G Connectivity for Immersive Digital Healthcare

The Internet of Things (IoT) allows interactions between patients and medical systems through real‐time patient data monitoring.^[^ ^17^, ^29^, ^163^, ^164^ ^]^ IoT technology can be combined efficiently with metaverse wearables, but immersive healthcare applications require fast feedback because the applications need to connect visual information beyond existing IoT health data.^[^ ^165^ ^]^ The 5G system helps to connect the interactions through networking feedback within 1 ms in smart health applications, and the cost of deployment and maintenance is lower than that of previous‐generation networks.^[^ ^166^, ^167^ ^]^

**Figure 12:**
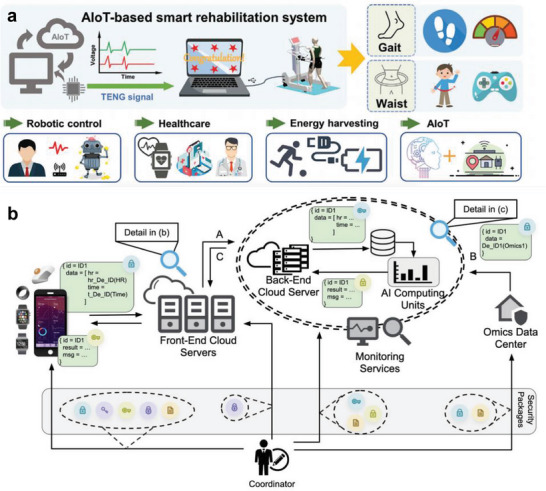
IoT and 5G Connectivity for digital healthcare. a) Schematic illustrations of an IoT‐based smart rehabilitation system for gait analysis and waist motion. AI algorithms and robots increase rehabilitation efficiency. Reproduced with permission.^[^ ^87^ ^]^ Copyright 2022, Wiley. b) Diagram of a personal health dashboard (PHD) platform to enhance data scalability, security, and interoperability. All data is encrypted through security packages. Reproduced with permission.^[^ ^168^ ^]^ Copyright 2021, Nature Portfolio.

An artificial IoT‐based rehabilitation system was introduced to increase the efficiency of long‐term rehabilitation (**Figure** **12**a).^[^ ^87^ ^]^ The system offers robot‐aided rehabilitation through artificial neural network‐based predictive modeling, which is trained by the personalized information acquired from sensors. The information obtained from the sensor was transferred to the cloud for analysis and can be visualized through the IoT system.

A personal health dashboard (PHD) platform has been developed to solve the challenges of IoT healthcare, including scalability, security, and interoperability (Figure 12b).^[^ ^168^ ^]^ IoT healthcare contains personalized data; thus, there is potential for threats to privacy and security regulations when the data are stored and accessed. The PHD platform can protect personalized information within a high‐performance computing system or large‐scale cloud to meet the needs of various end users. The registered information is encrypted and transferred to the data center to increase the security of personal data, and the encrypted data can interact with acquired wearables data. Continued development of IoT and network connectivity technology and information storage security is required to achieve latency‐free metaverse platforms.

## 4 Future Outlook

Recent technologies for connecting the metaverse and real space have continued to be developed, increasing the sense of immersion in virtual experiences. In addition, wearable devices deliver enhanced immersion in various experiences through haptic‐feedback and visual perception and offer numerous possibilities for healthcare applications through intelligent analysis and efficient sensing data processing.^[^ ^169^, ^170^, ^171^, ^172^ ^]^ However, despite these technological advances and efforts, some practical challenges and obstacles remain, and opportunities to improve immersion exist.

### 4.1 Broadening Connectivity for Collaboration with Various Fields

Due to the advent of the COVID‐19 pandemic, the interest in VR has increased in response to restrictions on social activities.^[^ ^173^ ^]^ In addition, the roles that can be performed in the metaverse are continuously increasing due to the development of digital financial technologies such as blockchain and virtual currency.^[^ ^174^ ^]^ However, the difference between the metaverse and XR is currently unclear because the metaverse platform is mainly implemented through XR technologies. Expansion to various fields of the metaverse is an anti‐discrimination strategy to increase the usability of the metaverse and share various experiences.^[^ ^3^ ^]^

The metaverse technology can perform roles beyond physical reality and provide convenience in reality activities such as SNS and education simultaneously.^[^ ^175^ ^]^ For example, the metaverse interface can offer private offices, realistic games, real estate, and business platforms. Sustainable services to users are an important factor for expanding the metaverse, and the key to sustainability is convenient interfaces, seamless connectivity, and high usability. Advanced wearable devices, fast communication, and integration between various fields are required to meet these requirements.

### 4.2 Visual Augmentation through Imaging Modalities

A camera system is crucial to construct virtual platforms for XR. Camera arrays or a single multi‐aperture camera system are required to acquire information on 3D objects or spaces, enhancing immersion in VR.^[^ ^176^, ^177^, ^178^ ^]^ However, conventional camera arrays or multi‐aperture camera systems are bulky, limiting their use in medical fields such as endoscopes.^[^ ^179^, ^180^ ^]^ A miniaturized multi‐aperture camera is required to explore the inside of narrow body structures to reconstruct 3D imaging for immersive laparoscopic surgery.^[^ ^181^, ^182^ ^]^ Biologically inspired cameras, such as an insect eye camera, can monitor an object with an ultra‐compact architecture. These camera systems are promising for acquiring 3D in vivo images for VR applications.^[^ ^120^, ^183^, ^184^ ^]^ The miniaturized camera is also advantageous for obtaining real‐time spatial information by combining it with a wearable device such as smart glasses. A multispectral camera beyond the visible light can offer information that is difficult to observe with the human eyes, so multispectral data acquired by the multispectral camera can provide enhanced experiences through augmented applications.

### 4.3 AI‐Generated Virtual Images

A recent innovative approach to creating images using only text commands to AI algorithms is receiving much attention.^[^ ^185^, ^186^, ^187^, ^188^, ^189^ ^]^ The innovation can generate images with custom visual characteristics such as gestures, backgrounds, and colors through intuitive and straightforward commands based on the learned data. However, only images in 2D space are provided with current technology, and the approach is limited in depicting 3D VR information. Nevertheless, AI can predict 3D structural information from 2D images, and AI‐generated 3D images can improve the sense of immersion in a virtual world. Also, an AI system can offer various surgical scenarios and decisions to improve efficiency in training and treatment.

### 4.4 Limitations of Long‐Term Power Supply

A continuous and stable power supply is essential to monitor and transmit data constantly. Despite recent advances in battery efficiency, they are still limited by bulky sizes and a short‐duration energy supply to drive wearable devices. The all‐solid‐state battery is a promising candidate due to its advantages of high energy density, long lifespan, and improved safety, but it has not yet been commercialized.^[^ ^190^, ^191^, ^192^ ^]^ In addition, the development of energy harvesting modules or wireless energy transmission can reduce the inconvenience of charging, but there still are limitations in wearable driving devices.^[^ ^193^, ^194^ ^]^

### 4.5 Brain‐Computer Interface Coupled with XR

Combining brain–computer interface (BCI) and XR can provide a communication channel and improve immersion in virtual space.^[^ ^195^, ^196^, ^197^ ^]^ BCI with XR technology delivers immersive scenarios by inducing illusions of artificially perceived reality and can smoothly adjust the intensity and complexity of stimuli.^[^ ^198^ ^]^ Communication through brain signals in virtual space can efficiently drive an avatar by improving the correspondence between the intention and response behavior, and the approach helps patients with movement disorders such as stroke. Precise brain signal acquisition and classification are required to improve the reliability of BCI further, and enhanced sensor arrays are also desired for acquiring various brain data. In addition, BCI with XR technology can contribute to mental therapy by analyzing the individual's psychological state and alleviating psychological pain through contextual videos.^[^ ^199^, ^200^ ^]^

### 4.6 Data Encryption for Privacy Protection

Data security is critical in healthcare because medical history or personal health data contain permanent valuable information. In particular, medical information must be encrypted and preserved according to the Health Insurance Portability and Accountability Act.^[^ ^201^, ^202^ ^]^ A data transmission process is essential because the bio‐signals from wearable devices should be analyzed after being delivered to a cloud computing system. The data can be leaked or damaged during transmission, and incorrect feedback may be fatal to patients. The health data should be encrypted and analyzed, but the procedures can induce a time delay that prevents immediate feedback.^[^ ^203^ ^]^ The encryption and analysis algorithms must be optimized to minimize feedback delay, or personalized signal processing can be performed through a neuromorphic circuit.^[^ ^204^, ^205^ ^]^

### 4.7 Practice Assistance for Maternal Delivery in XR‐Based Education

As a special case, in the Republic of Korea, which has registered the world's lowest birth rate, encountering childbirth cases during hospital internships in clinical settings poses a significant challenge.^[^ ^206^ ^]^ This difficulty is further compounded by patient rights and privacy issues, making it increasingly difficult for medical students to gain exposure to childbirth cases. Leveraging metaverse wearable technology, we suggest that even challenging medical scenarios that are rarely encountered in a clinical context can be repeatedly simulated. This approach effectively enhances clinical capabilities in a dynamic, immersive, and ethically considerate learning environment.

### 4.8 Physical Monitoring of Patients with Chronic Disease and Elderly Individuals in the Metaverse

Avatar‐based therapeutic and nursing practices in a metaverse environment can be utilized effectively for monitoring and managing the health of chronic disease patients and elderly individuals, aligning with the requirements of a rapidly aging/aged society. It is imperative to incorporate metaverse wearables that account for comfort and safety for elderly individuals, specifically designed to mitigate risks such as falls or dizziness during prolonged use.

Using metaverse wearables, seniors can engage in fitness activities in a virtual environment, mitigating feelings of isolation.^[^ ^207^, ^208^ ^]^ Wearable devices allow the monitoring of vital signs, linking patients with medical professionals for effective health management. Engaging in virtual physical activities such as walking, yoga, and gymnastics, coupled with real‐time feedback, facilitates proactive health management. Seniors can maintain their physical activity and uphold a healthy lifestyle together. This enables the creation of social interaction and virtual communities. Specifically for elderly individuals, experiencing social interaction through avatars in the metaverse fosters participation in virtual communities. This affords opportunities for conversation and sharing interests with others. Such an approach is poised to reduce social isolation among seniors, enhancing communication and fostering social connectivity.

Moreover, metaverse wearables can be a supportive tool for cognitive training and brain activity for elderly individuals. As environments that facilitate familiarity with smartphones and digital virtual environments become accessible, memory‐based games, virtual problem‐solving, and cognitive training can be undertaken. This promotes the maintenance and enhancement of cognitive abilities for seniors, enabling them to sustain cognitive function and promote brain health. This approach may dovetail with brain neuromorphic electronics in the future, further enhancing immersive experiences.

### 4.9 Pandemic Management for Preparedness of Infectious Diseases

Last, metaverse wearables can be employed to manage infectious disease pandemics. Utilizing metaverse platforms combined with XR technology to design and construct a virtual world for pandemic preparedness is valuable. Leveraging this virtual space can support social distancing measures, personal hygiene management, and infectious disease prevention activities in a metaverse environment. Therefore, we believe this approach can potentially help find a new way to reinforce response capabilities during pandemic situations.

## 5 Summary

Recent advances in metaverse and XR technology offer innovative experiences in the healthcare field, including surgical training, rehabilitation, and treatment. The combination of XR wearable devices and metaverse technology can foster a sense of immersion and improve quality of life through advances in various healthcare fields. Wearable devices mounted on hands, arms, feet, body, face, etc., acquire various signals to analyze gestures or gaits, and the acquired signals are connected with the virtual world to deliver immersive experiences. Future applications of generative or neural AIs and ultrafast internet network technology are possibly required for further advancements into metaverse technology, which provides enhanced experiences in immersive digital healthcare applications to reflect personal preferences. Furthermore, we believe that metaverse wearables coupled with XR technology will also contribute to realizing a paradigm shift in everyday life by being applied to various healthcare fields, such as personalized occupational, educational, and home healthcare applications in the post‐pandemic era.

## Conflict of Interest

The authors declare no conflict of interest.

## Author Contributions

K.K. and W.G.L. conceived the idea. K.K. curated the existing data. K.K., H.Y., and W.G.L. wrote the main contents. K.K., H.Y., and W.G.L. designed the research search scheme. K.K. and J.L. decorated the figure. K.K. and W.G.L. supervised the manuscript, and all authors edited and wrote the final version of the manuscript.
